# Additives Enhancing the Catalytic Properties of Lipase from *Burkholderia cepacia* Immobilized on Mixed-Function-Grafted Mesoporous Silica Gel

**DOI:** 10.3390/molecules19079818

**Published:** 2014-07-08

**Authors:** Emese Abaházi, Zoltán Boros, László Poppe

**Affiliations:** 1 Department of Organic Chemistry and Technology, Budapest University of Technology and Economics, Műegyetem rkp. 3, Budapest H-1111, Hungary; E-Mails: abahazi.emese@mail.bme.hu (E.A.); zoltan.boros@synbiocat.com (Z.B.); 2 SynBiocat LLC., Lázár deák u. 4/1, Budapest H-1173, Hungary

**Keywords:** silica gel, *Burkholderia cepacia* lipase, enzyme immobilization, adsorption, activity enhancement, additive, continuous-flow packed-bed reactor

## Abstract

Effects of various additives on the lipase from *Burkholderia cepacia* (*Bc*L) immobilized on mixed-function-grafted mesoporous silica gel support by hydrophobic adsorption and covalent attachment were investigated. Catalytic properties of the immobilized biocatalysts were characterized in kinetic resolution of racemic 1-phenylethanol (*rac***-1a**) and 1-(thiophen-2-yl)ethan-1-ol (*rac***-1b**). Screening of more than 40 additives showed significantly enhanced productivity of immobilized *Bc*L with several additives such as PEGs, oleic acid and polyvinyl alcohol. Effects of substrate concentration and temperature between 0–100 °C on kinetic resolution of *rac***-1a** were studied with the best adsorbed *Bc*Ls containing PEG 20 k or PVA 18–88 additives in continuous-flow packed-bed reactor. The optimum temperature of lipase activity for *Bc*L co-immobilized with PEG 20k found at around 30 °C determined in the continuous-flow system increased remarkably to around 80 °C for *Bc*L co-immobilized with PVA 18–88.

## 1. Introduction

The interest in use of enzymes as natural chiral catalysts has increased in the past few decades [1,2,3,4]. Chirality has become a central topic in pharmaceutical industry [5,6,7], thus the application of enzymes as biocatalysts in the production of enantiopure chiral compounds turned out to be relevant on industrial scale [8]. Hydrolases, especially lipases, are the most often used biocatalysts in asymmetric biotransformations, because they can catalyze a wide range of enantio- and regioselective reactions such as hydrolysis, esterification, transesterification, aminolysis and ammoniolysis [9,10,11].

Lipases (triacylglycerol hydrolases, EC 3.1.1.3) catalyzing the hydrolysis of the triglycerides into fatty acids, mono-, and diacylglycerols, and glycerol [12] at the lipid-water interface [13,14] belong to the enzyme class of hydrolases. Because lipases are relatively thermostable and often highly selective in their reactions with a wide range of substrates, they are widely used in food, detergent and pharmaceutical industry [8,9,10].

In spite of their enormous synthetic potential, the application of enzymes as native proteins has some drawbacks. Many enzymes are relatively unstable in aqueous solutions and their recycling is difficult. For industrial applications, immobilization of enzymes proved to be crucial to enhance their activity, thermal and operational stability, and reusability [15,16,17,18]. Among the numerous methods developed for enzyme immobilization [15,16,17,18], including adsorption, covalent attachment to solid supports and entrapment within polymers, immobilization of the biocatalysts onto solid supports has become a robust, widely accepted industrial technique [19,20,21]. Physical adsorption of the desired enzyme onto suitable carriers is a convenient, one step immobilization technique, especially for lipases [15,16,17,18,19,20,21,22].

The nature of the solid support in enzyme immobilization is of primary importance [20,23]. Porous silica gels [24], particularly mesoporous silica gels (MPSs) [25,26,27] turned out to be useful carriers for enzyme immobilization due to their large surface area, tunable porosity, low cytotoxicity, favorable mechanical properties and functionalizable large surface. Surface grafting of MPSs with variable functions can widen their applicability as carriers for proteins and enzymes [25,27]. Surface-functionalized silica gels, such as butyl [28] or octyl silica gels [29] proved to be suitable carriers for adsorptive immobilization of lipases [30]. Furthermore, hydrophobic silica gels were useful for differential adsorption of lipase A and lipase B from *Candida antarctica* [31]. Mixed-function-grafted silica supports with amine groups allowed the immobilization of enzymes by adsorption as well as by covalent immobilization [32].

Lipase catalysis is characterized by interfacial activation. When lipases are dissolved in water, their active site is covered by a lid resulting in a closed, catalytically inactive form. When lipases are in contact with an interface between water and apolar phase, the lid opens allowing access to the active site [12,13,14]. The increased hydrophobicity near the active site in the open conformation is the basis of preferential adsorption and interfacial activation of lipases during adsorption on hydrophobic surfaces [16,17,18]. Because of this conformational mobility influencing the catalytic activity, the final outcome of the biocatalytic properties of immobilized lipases can be influenced by various additives during adsorption and covalent attachment.

Molecular imprinting [33] – which is called bioimprinting when enzymes are tuned at their active site by substrates or their analogues – proved to be one of the most successful strategies for enhancing enzyme activity in organic solvents [34,35,36,37,38,39]. The active site of the lipase treated with substrate analogues, surfactants or other entities, resulted in improved lipase performance in non-aqueous medium [40,41,42,43]. Conformational changes opening the lid over the active site occur during the bioimprinting process and thus the immobilized lipase is fixed in an open conformation. When the ligand is washed away, the enzyme is trapped in this conformation because it has adopted a rigid structure due to the strong intramolecular electrostatic interactions that occur in a solvent with a low dielectric constant [44,45]. Combining molecular imprinting with protein surface coating and salt activation was reported as dual bioimprinting [42].

Biodegradable polymers such as polyvinyl alcohol (PVA) or chitosan were applied as additives in enzyme immobilizations [46]. Further ecofriendly polymers such as gum arabic and chitosan were also useful for stabilization of enzymes by microencapsulation [47]. In addition, PVAs were applied in preparation of sol-gel catalysts as lipase stabilizing additives [48,49]. The beneficial effects of further additives such as crown ethers, β-cyclodextrin derivatives, surfactants and sugars were also studied in sol-gel encapsulations [48,50,51].

The biotransformations may not only be tuned by modifying the biocatalysts but also by the reaction conditions. Effect of the temperature on selectivity is one of the major concerns in enzymatic transformations. In most of the cases – usually investigated in batch mode – stereoselectivity of enzyme catalyzed reactions was decreased with increasing temperature [52,53,54,55,56,57,58,59,60,61,62]. A few examples were found where enantiomer selectivity of enzyme-catalyzed kinetic resolutions (KRs) increased with increasing temperature [63,64] or had a maximum [32,65,66]. Although biotransformations in continuous-flow systems could enhance the efficiency of the hydrolase-catalyzed processes [67], there are only a few examples on the temperature effects on lipase-catalyzed KRs in continuous-flow mode so far [32,51,65,66,67,68].

Lipase from *Burkholderia cepacia* (*Bc*L) was selected to study the effect of different types of additives during immobilization on surface-grafted mesoporous silica gels on enhancing the enzymatic activity and selectivity. *Bc*L is an extracellular lipase catalyzing the biodegradation of environmental pollutants, biological control of plant diseases [69]. *Bc*L being a relatively thermotolerant enzyme is frequently used as biocatalyst in various biotransformations performed in non-aqueous media [50,70,71,72]. In addition to study the influence of wide range of additives on the biocatalytic properties of *Bc*L immobilized onto surface modified silica gels, the temperature effect on the immobilized *Bc*L-catalyzed KRs in continuous-flow mode was also investigated.

## 2. Results and Discussion

### 2.1. Immobilization of *Bc*L onto Mixed-Function-Grafted Mesoporous Silica Gels

In this study on immobilization of *Bc*L onto mixed-function-grafted mesoporous silica gel by adsorption and adsorption combined with covalent binding, more than 40 additives were tested as bioimprinting agents, stabilizers or surface coating polymers. The additives in our present study were mono- (rhamnose, glucose, fructose, xylitol, xylose, sorbitol, mannitol), di- (sucrose, maltose) and polysaccharides (xylane, gum arabic, carrageenan, sodium alginate, chitosan, α- and β-cyclodextrins, starch); polyethylene glycols (tetraethylene glycol, PEG 400, PEG 1k, PEG 4k, PEG 8k, PEG 20k, PEG 1k dimethyl ether); polyvinyl alcohols (PVA 4–88, PVA 18–88, PVA 13–23–88, PVA 60–98, PVA 72–98); detergents (Brij 30, Triton X-100, Tween 80); carboxylic and fatty acids (hexanoic, octanoic, decanoic, lauric, palmitic, oleic acid); glycerol and glycerides (glycerol, trilaurin, triolein, tristearin, Hymono 9004—a mixture of unsaturated mono- and diglycerides, FAME—fatty acids methyl esters) and unnatural substrates (1-phenylethanol, 1-(thiophen-2-yl)ethan-1-ol, 2-octanol).

### 2.2. Effect of Additives on the Biocatalytic Properties of Adsorbed *Bc*L

**Scheme 1:**
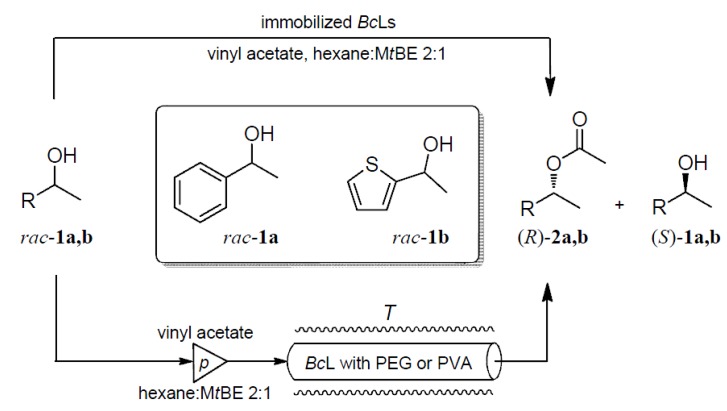
Kinetic resolutions of 1-phenylethanol (*rac***-1a**) and 1-(thiophen-2-yl)ethan-1-ol (*rac***-1b**) with differently immobilized *Bc*L biocatalysts in batch and continuous-flow modes.

Adsorption of *Bc*L onto mixed-function-grafted mesoporous silica gel from aqueous media was carried out in the presence of the additives listed in the Section 2.1. To evaluate the effects of the various additives on the biocatalytic properties of immobilized *Bc*L biocatalysts, the resulted *Bc*L preparations were investigated in the kinetic resolutions of racemic secondary alcohols 1-phenylethanol (*rac***-1a**) and 1-(thiophen-2-yl)ethan-1-ol (*rac***-1b**) in batch mode—in shake vials—(Table 1) and in continuous-flow bioreactors (Scheme 1).

**Table 1:** Biocatalytic properties of *Bc*L adsorbed onto mixed function-grafted silica supports in the kinetic resolution of *rac-***1a** and *rac***-1b** in batch mode.

Additive	KR of *rac*-1a ^a^				KR of *rac-*1b ^b^			
	*c* (%)	*ee*_(*R*)-**2a**_ (%)	*E*	*U*_b_ (µmol g^−1^ min^−1^)	*c* (%)	*ee*_(*R*)-**2b**_ (%)	*E*	*U*_b_ (µmol g^−1^ min^−1^)
-	2.3	98.1	>100	1.6	2.1	76.6	7.7	2.7
Brij 30	5.1	98.9	>100	3.5	4.2	74.3	7.0	5.5
Tween 80	9.9	99.2	>200	6.7	8.8	74.9	7.5	11.5
PVA 4–88	26.1	99.5	»200	17.9	27.5	75.0	9.2	35.9
PVA 18–88	22.5	99.4	>200	15.4	21.8	75.2	8.6	28.4
PVA 13–23–88	25.8	99.3	>200	17.6	27.2	76.4	9.8	35.4
PVA 72–98	14.8	98.9	>200	10.1	14.0	74.5	7.7	18.2
PEG 8k	24.6	99.5	»200	16.8	26.7	74.0	8.7	34.8
PEG 20k	30.0	99.0	>200	20.5	34.7	74.6	10.1	45.1
Gum arabic	21.8	99.3	>200	14.8	19.9	74.6	8.2	25.8
Chitosan	11.5	98.7	>100	7.9	9.8	74.9	7.6	12.8
Lauric acid	13.8	99.3	>200	9.4	9.9	74.0	7.2	12.8
Oleic acid	23.3	99.3	>200	15.9	14.8	73.0	7.3	19.2
Hymono 9004	22.5	99.5	»200	15.4	11.6	73.4	7.2	15.1
Trilaurin	14.2	99.4	>200	9.7	5.7	73.4	6.8	7.5
Triolein	23.5	99.5	»200	15.9	10.3	73.8	7.2	13.3
*rac***-1a**	10.8	99.0	>200	7.3	-	-	-	-
*rac***-1b**	-	-	-	-	5.3	72.1	6.4	6.8

^a^ The conversion (*c*) and enantiomeric excess of ester (*ee*_(*R*)-**2a**_) was determined by chiral GC and enantiomeric ratio (*E*) was calculated from *c* and *ee*_(*R*)-**2a**_. The results of KRs with immobilized *Bc*Ls are shown only for biocatalysts with *U*_b_ > 5.0 µmol g^−1^ min^−1^; ^b^ The conversion (*c*) and enantiomeric excess of ester (*ee*_(*R*)-**2b**_) was determined by chiral GC and enantiomeric ratio (*E*) was calculated from *c* and *ee*_(*R*)-**2b**_. The results of KRs with immobilized *Bc*Ls are shown only for biocatalysts with *U*_b_ > 3.0 µmol g^−1^ min^−1^. *Reaction conditions*: Adsorption on mixed-function-grafted silica gel (20.0 mg mL^−1^) in a mixture of Tris buffer (14.3 mL, 100 mM, pH = 7.5 ionic strength controlled with NaCl) and 2-propanol (750 µL), additive (2.0 mg mL^−1^) and *Bc*L (2.0 mg mL^−1^), 400 rpm, 4 °C, 24 h; KR of *rac***-1a**: *rac***-1a** (25.0 mg mL^−1^) and *Bc*L adsorbed on mixed-function-grafted silica gel (12.5 mg mL^−1^) in a mixture of hexane/*tert*-butyl methyl ether/vinyl acetate 6/3/1 (2.0 mL), 1000 rpm, 30 °C, 4 h; KR of *rac***-1b**: *rac***-1b** (25.0 mg mL^−1^) and *Bc*L adsorbed on mixed-function-grafted silica gel (12.5 mg mL^−1^) in a mixture of hexane/*tert*-butyl methyl ether/vinyl acetate 6/3/1 (2.0 mL), 1000 rpm, 30 °C, 2 h (see Experimental)

Data from KRs of racemic 1-phenylethanol (*rac***-1a**) (Table 1) showed that several additives enhanced significantly the catalytic properties of the adsorbed *Bc*L. While the conversion without additive was only 2.3% (*U*_b_ = 1.6 µmol g^−1^ min^−1^; *E* > 100 ) after 4 h of KR of *rac***-1a**, all the additives listed in Table 1 improved the productivity of adsorbed *Bc*L. Remarkably, the enantiomer selectivity of *Bc*L increased in each case as well. While sugars and monosaccharides had no significant impact on activity of *Bc*L (data not shown), the surfactant Tween 80 enhanced slightly the activity of *Bc*L (*U*_b_ = 6.7 µmol g^−1^ min^−1^; *E* > 100). The natural substrates of lipases such as trilaurin and triolein resulted in a greater enhancement of activity (e.g., triolein increased the specific activity by 10-fold). The highest activity enhancements were achieved with PVAs and polyethylene glycols. Among the PVAs tested, addition of PVA 4–88 resulted in the most significant 11-fold activity enhancement (*U*_b_ = 17.9 µmol g^−1^ min^−1^; *E* » 200). The most active adsorbed *Bc*L/PEG 20k biocatalyst had 13-fold higher enzyme activity in acylation of *rac***-1a** (*U*_b_ = 20.5 µmol g^−1^ min^−1^; *E* > 200) than *Bc*L adsorbed without any additives. Although racemic 1-phenylethanol *rac-***1a** is not an ester or carboxylic acid-type substrate, the 2.5-fold activity enhancement of the *rac-***1a**-treated *Bc*L compared to the non-treated *Bc*L preparation in KR of *rac-***1a** indicated significant bioimprinting effect.

The best results of KRs of racemic 1-(thiophen-2-yl)ethan-1-ol (*rac***-1b**) with the adsorbed *Bc*L biocatalysts treated with the additives are shown in Table 1. The *Bc*L adsorbed without any additive resulted in only 2.1% conversion after 2 h (*U*_b_ = 2.7 µmol g^−1^ min^−1^; *E* = 7.7). Similarly to the KRs of 1-phenylethanol (*rac***-1a**), surfactants enhanced the specific enzyme activity in KRs of *rac***-1b** by 2- and 4-fold (*U*_b_ = 5.5 and 11.5 µmol g^−1^ min^−1^; with Brij 30 and Tween 80, respectively). The natural substrates (triolein, trilaurin, oleic acid and lauric acid) as additive showed higher imprinting effects with the adsorbed *Bc*L (2.8–7.1-fold increase of *U*_b_). In the same way as in the KRs of *rac***-1a**, the most active *Bc*L biocatalysts in KRs of 1-(thiophen-2-yl)ethan-1-ol *rac***-1b** were the ones adsorbed in the presence of PVAs and polyethylene glycols as additives (13-fold enhancement, *U*_b_ = 35.9 µmol g^−1^ min^−1^ and 17-fold activity enhancement, *U*_b_ = 45.1 µmol g^−1^ min^−1^; with PVA 4–88 and PEG 20k, respectively). Expectedly, in KR of *rac-***1b** the *rac***-1b**-treated *Bc*L showed more significant bioimprinting effect (2.4-fold increase in *U*_b_ compared to the non-treated *Bc*L preparation) than the *rac-***1a**-treated one (less than 2-fold enhancement of *U*_b_).

### 2.3. Effect of Additives on the Biocatalytic Properties of Covalently Immobilized *Bc*L

A further goal was to combine the benefits of adsorption and covalent binding onto mixed-function-grafted silica gel with amino functions and to further improve in this way the stability of the covalently-linked *Bc*L. It has been shown that heterofunctional supports can be advantageous for enzyme immobilization from multiple points of view [73]. Because phenyl grafting on silica gel was beneficial for hydrophobic adsorption and lipase activation [30,31] and covalent immobilization of *Ca*LB on amino-silica resulted in better thermal stability of the enzyme than simple physical adsorption [32,74], it was assumed that adsorption on an amino-phenyl mixed-function-grafted silica gel support followed by cross-linking may provide a more stable cross-linked *Bc*L that is also attached at the same time to the support by covalent bonds as well. Thus, a mesoporous silica gel (Dv250) grafted with (3-aminopropyl)trimethoxysilane (APTMOS) and phenyltrimethoxysilane (PTMOS) at 1:3 ratio which allowed the efficient immobilization of lipase B from *Candida antarctica* by adsorption and covalent cross-linking [32] was selected as carrier to perform the adsorption and covalent immobilization of *Bc*L in the presence of seven well performing additives (PEG 4k, Tween 80, PVA 18–88, gum arabic, triolein, lauric acid and oleic acid). However, based on our recent results with glycerol diglycidyl ether (GDE) as an efficient cross-linker for preparation of cross-linked enzyme aggregates [75], GDE was applied as cross-linking agent instead of the previously used glutardialdehyde [32]. As it was already shown, GDE may be particularly useful as cross-linking agent due to its ability to form stable bonds under mild conditions not only with the amine groups of Lys but with sulfur and oxygen containing residues of Cys, Tyr, Glu or Asp as well [75]. Furthermore, GDE is an inexpensive, partially water soluble bis-epoxy compound being less toxic than glutaraldehyde (GA).

**Figure 1:**
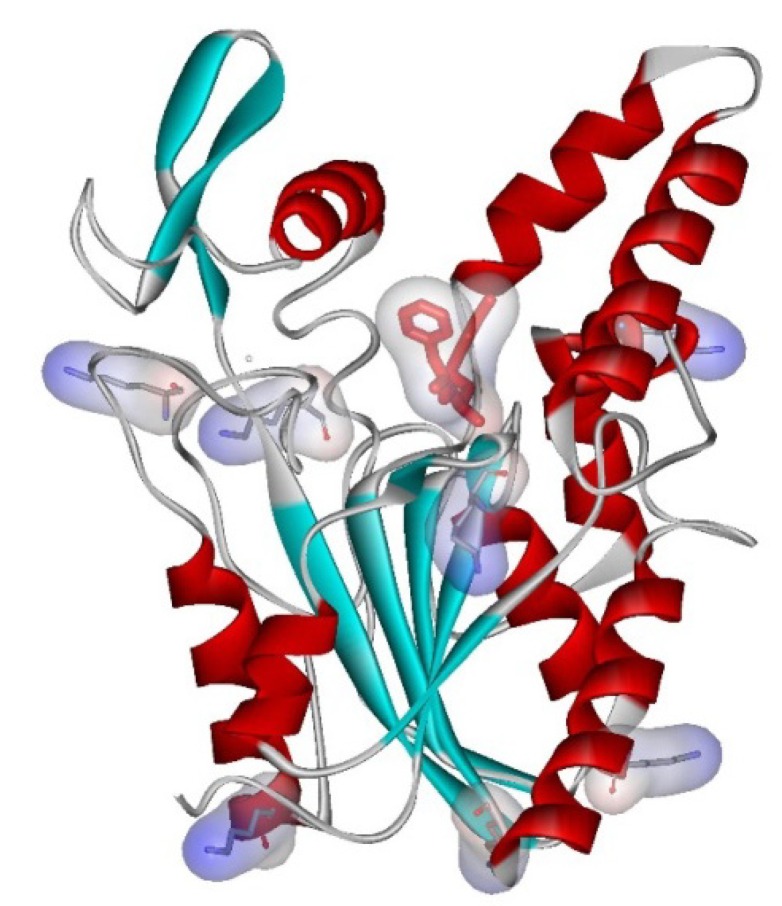
The catalytically active open conformation of *Bc*L (PDB code: 1YS1 [76]) with a substrate analogue (in red) and the surface exposed Lys residues (in CPK color).

The results of GDE cross-linking after adsorption of *Bc*L without additives were discouraging because the resulted *Bc*L preparations exhibited almost no activity in KRs of *rac***-1a** and *rac***-1b** (only 0.3% and 0.5% conversions after 24 h, respectively). The results may be rationalized by taking the spatial distribution of the surface exposed Lys residues in the open conformation of *Bc*L into account (Figure 1). The structure shown in Figure 1 clearly indicates that the majority of the surface exposed Lys residues (four out of seven) are close to the lid domains modulating the active site accessibility. Thus anchoring the enzyme by these residues may force—at least partially—the closure of the entrance to the active site leading to inactive forms of the immobilized *Bc*L.

Fortunately, all the eight selected additives resulted in enhancement of the biocatalytic properties of the cross-linked *Bc*L preparations (Table 2). Natural carboxylic acid substrates such as lauric acid and oleic acid applied as additives during cross-linking enhanced the biocatalytic properties of *Bc*L (almost tenfold increase of activity). Addition of larger ester-containing additives such as triolein or Tween 80 further enhanced the conversion (*c* = 7.3 and 8.7%, respectively). The best results were achieved with large molecular weight polymeric additives such as PEG 20k (*c* = 11.8%), gum arabic (*c* = 13.5%) and PVA 18–88 (*c* = 14.0%). The beneficial effect may be rationalized by the lower hydrophobicity of the polymeric additives forcing opposite orientation at the surface of the carrier and rigidification of the enzyme in proper conformation during the cross-linking process.

**Table 2:** Biocatalytic properties of *Bc*L covalently attached by GDE cross-linker onto mixed-function-grafted mesoporous silica gel in the kinetic resolution of *rac***-1a** and rac**-1b** in batch mode.

	KR of *rac*-1a ^a^			KR of *rac*-1b ^b^		
Additive	*c* (%)	*ee*_(*R*)-**2a**_ (%)	*E*	*c* (%)	*ee*_(*R*)-**2b**_ (%)	*E*
-	0.3	89.8	18.6	0.5	70.4	5.8
PEG 4k	1.2	97.9	94.8	1.3	71.4	6.1
PEG 20k	11.8	99.5	»200	22.3	73.3	8.2
Tween 80	7.3	99.6	»200	9.4	71.6	6.5
PVA 18–88	14.0	99.8	»200	26.4	75.2	9.2
Gum arabic	13.5	99.8	»200	26.1	75.5	9.3
Lauric acid	4.0	99.4	>200	8.5	74.7	7.4
Oleic acid	3.4	99.4	>200	9.9	74.8	7.5
Triolein	8.7	99.7	»200	13.0	73.3	7.2

^a^ The conversion (*c*) and enantiomeric excess of ester (*ee*_(*R*)-**2a**_) was determined by chiral GC and enantiomeric ratio (*E*) was calculated from *c* and *ee*_(*R*)-**2a**_. ^b^ The conversion (*c*) and enantiomeric excess of ester (*ee*_(*R*)-**2b**_) was determined by chiral GC and enantiomeric ratio (*E*) was calculated from *c* and *ee*_(*R*)-**2b**_. *Reaction conditions*: Cross-linking: GDE (20 µL mL^−1^), additive (1.9 mg mL^−1^) and *Bc*L adsorbed on mixed-function-grafted silica gel (12.5 mg mL^−1^) in a mixture of phosphate buffer (4 mL, 20 mM, pH = 7.2) and ethanol (12 mL), 400 rpm, 25 °C, 24 h; KR of *rac***-1a**: *rac***-1a** (25.0 mg mL^−1^) and *Bc*L adsorbed on mixed-function-grafted silica gel (12.5 mg mL^−1^) in a mixture of hexane/*tert*-butyl methyl ether/vinyl acetate 6/3/1 (2.0 mL), 1000 rpm, 30 °C, 24 h; KR of *rac***-1b**: *rac***-1b** (25.0 mg mL^−1^) and *Bc*L adsorbed on mixed-function-grafted silica gel (12.5 mg mL^−1^) in a mixture of hexane/*tert*-butyl methyl ether/vinyl acetate 6/3/1 (2.0 mL), 1000 rpm, 30 °C, 24 h (see Experimental).

The biocatalytic properties of the *Bc*L preparations cross-linked in the presence of the eight selected additives were also tested in the KR of *rac***-1b** (Table 2). Similarly to the KR tests with *rac-***1a**, all the eight covalently bound *Bc*L biocatalyst prepared in the presence of additives showed enhanced biocatalytic features in the KRs with *rac-***1b** as well. PEG 4k resulted in 2.6-fold increase in conversion, the bioimprinting additives caused even larger effect (17- to 26-fold increase of the conversion with lauric acid, Tween 80, oleic acid and triolein). The most significantly enhanced conversions in the KRs with *rac-***1b** with covalently immobilized *Bc*L were found by using the large molecular weight polymeric additives PEG 20k, gum arabic and PVA 18–88 (45- to 53-fold increase).

Although use of proper additives during the cross-linking of the adsorbed *Bc*L enhanced the biocatalytic properties of the covalently immobilized *Bc*L, the specific activity (*U*_b_) of the adsorbed and cross-linked *Bc*L preparations remained only 5%–15% that of the adsorbed *Bc*L biocatalysts. Due to the simplicity of the adsorption process and to the tenfold higher specific activity of the adsorbed *Bc*L, only the adsorbed lipase biocatalysts were investigated further.

### 2.4. Thermal Stability of *Bc*Ls Adsorbed onto Mixed-Function-Grafted Mesoporous Silica Gel

**Figure 2:**
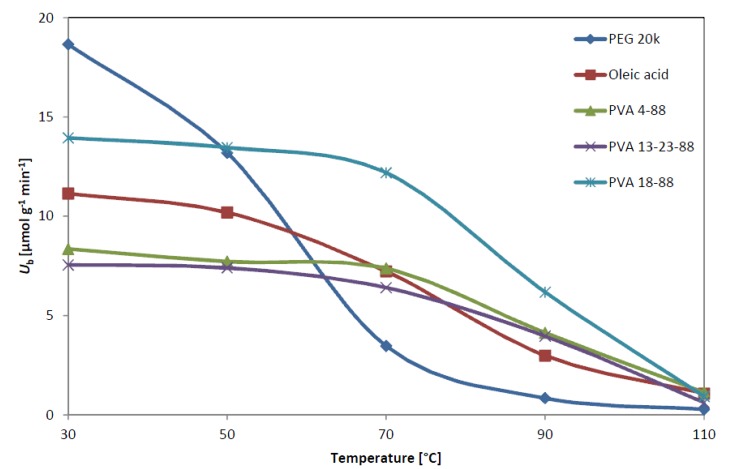
Thermal stability of adsorbed *Bc*L preparations in kinetic resolution of *rac***-1a**.

Five immobilized *Bc*Ls adsorbed onto mixed-function-grafted mesoporous silica gel in the presence of additives with the highest *U*_b_ with *rac***-1a** (see Table 1) were selected for the further study on thermal stability of the adsorbed *Bc*Ls. During the thermostability tests, the *Bc*L samples adsorbed in the presence of the five selected additives were incubated in toluene at various temperatures (30, 50, 70, 90 and 110 °C) for 1 h and were tested in KR of *rac***-1a** after cooling to 30 °C. Figure 2 shows the specific activity of the five selected *Bc*Ls at given temperatures in the KRs after 2 h.

*Reaction conditions*: Thermal treatment: *Bc*L adsorbed on mixed-function-grafted silica gel (25 mg) in toluene (1 mL), 1 h at the indicated temperature; KR test with *rac***-1a**: *rac***-1a** (25.0 mg mL^−1^) and *Bc*L adsorbed on mixed-function-grafted silica gel (12.5 mg mL^−1^) in a mixture of hexane/*tert*-butyl methyl ether/vinyl acetate 6/3/1 (2.0 mL), 1000 rpm, 30 °C, 2 h (see Experimental).

According to the KR tests with *rac***-1a** at 30 °C, the most active adsorbed *Bc*L preparation was the one obtained with PEG 20k as additive (*U*_b_ = 18.7 µmol g^−1^ min^−1^). The *Bc*L/PEG 20k form, however, proved to be the least thermostable (retained only 18% of the initial activity after incubating at 70 °C for 1 h). When tested at 30 °C, the second most active form of adsorbed *Bc*L was the one prepared with PVA 18–88 as additive (*U*_b_ = 14.0 µmol g^−1^ min^−1^). The *Bc*L / PVA 18–88 form turned out to be one of the most thermostable preparations (87% of the initial activity was retained after incubating at 70 °C). While the high molecular weight PVA 18–88 (130 kDa, 88% hydrolyzed) or PVA 4–88 (31 kDa, 88% hydrolyzed) as additive had almost the same relative stabilizing effect for *Bc*L up to 70 °C, the less molecular weight PVA 13–23–88 (13–23 kDa, 88% hydrolyzed) resulted in lower degree of thermal stabilization (only up to 50 °C). Oleic acid as natural substrate for *Bc*L showed even less thermal stabilization. The most thermostable forms of *Bc*L with PVA 4–88 and PVA 18–88 retained 44 and 49% of their initial activity after 1 h incubation at 90 °C but all *Bc*L forms lost their activity after incubating at 110 °C. The thermal behavior of adsorbed *Bc*L with PEG 20k can be rationalized by assuming that enhanced thermostability is related to embedding the lipase molecule within a thin film of the high molecular weight additive which is diminished when PEG 20k melts (Mp ~ 60 °C).

Considering also the fact that the more hydrophilic, almost fully (98%) hydrolyzed PVAs were much less active (*U*_b_ = 10.1 µmol g^−1^ min^−1^ with PVA 72–98: 72 kDa; and *U*_b_ < 5 µmol g^−1^ min^−1^ with PVA 60–98: 60 kDa; see Table 1) than PVAs with 88% hydrolysis (*U*_b_ = 15.4–17.9 µmol g^−1^ min^−1^ with PVA 18–88: 130 kDa, PVA 4–88: 31 kDa and PVA 13–23–88: 13–23 kDa; see Table 1), it can be assumed that partial hydrophobicity of less hydrolyzed PVAs forming a thin film embedding the lipase molecules contributed to the activation of *Bc*L.

### 2.5. Recyclability of Adsorbed *Bc*L Biocatalysts

**Figure 3:**
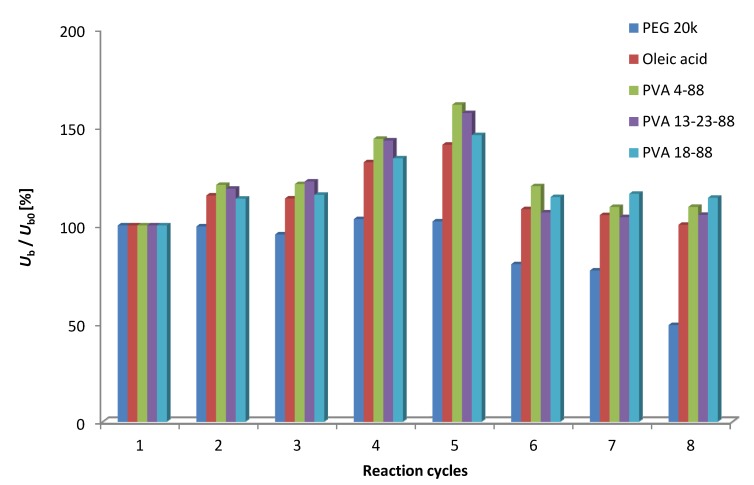
Recycling of the adsorbed *Bc*L from repeated kinetic resolutions of *rac***-1a**.

For practical applications of immobilized lipases, reusability is of foremost importance. For testing the operational stability and recycling of the *Bc*Ls adsorbed onto mixed-function-grafted mesoporous silica gel with additives, the five adsorbed *Bc*L biocatalysts with the highest specific biocatalyst activity (*U*_b_) in KR of *rac***-1a** were compared by repeated KRs of *rac***-1a** as recycling test. Each biocatalyst was reused seven times and their *U*_b_ (initial values are listed in Table 1) was recorded after recovery. Figure 3 shows the retained relative specific activities of adsorbed *Bc*L biocatalysts related to their initial specific biocatalyst activity as 100%.

Recycling of the adsorbed *Bc*L biocatalysts in eight cycles of KR of *rac***-1a** indicated different effect of the additives on the operational stability of the adsorbed *Bc*L in organic solvent. Four out of the investigated five additives (oleic acid and three PVAs) resulted in quite stable adsorbed *Bc*L biocatalysts retaining their initial specific biocatalyst activity even after eight runs. In contrast, the *Bc*L biocatalyst adsorbed in the presence of PEG 20k lost gradually its activity and retained only 49% of its initial *U*_b_ after eight runs. The apparent increase of the initial *U*_b_ which may be rationalized by equilibration/partial loss of water content of the enzyme during repeated runs is noteworthy. Due to the good mechanical properties of carrier, the mass loss of biocatalysts was below 5% in each cycle.

*Reaction conditions for one cycle*: *rac***-1a** (25.0 mg mL^−1^) and *Bc*L adsorbed on mixed-function-grafted silica gel (12.5 mg mL^−1^) in a mixture of hexane/*tert*-butyl methyl ether/vinyl acetate 6/3/1 (2.0 mL), 1000 rpm, 30 °C, 1 h; then washing by hexane (2 × 10 mL). For GC after each cycles, see Experimental.

### 2.6. Continuous-Flow Kinetic Resolutions of rac**-1a** with *Bc*Ls Adsorbed with PVA 18-88 and PEG 20k

Retention of biocatalysts by immobilization—in particular for enzymes [15,16,17,18,19,20,21], especially for lipases [22]—contributed significantly to the development of continuous-flow biotransformations [8,19,67]. When a biotransformation is performed in a continuous-flow packed-bed bioreactor, no separate process is required for enzyme recovery because the immobilized biocatalyst is continuously retained. Moreover, packed-bed flow reactors with immobilized catalyst have a clear advantage in that voidage is low: 34% compared to over 80%–90% being typical for a stirred tank reactor [77].

**Figure 4:**
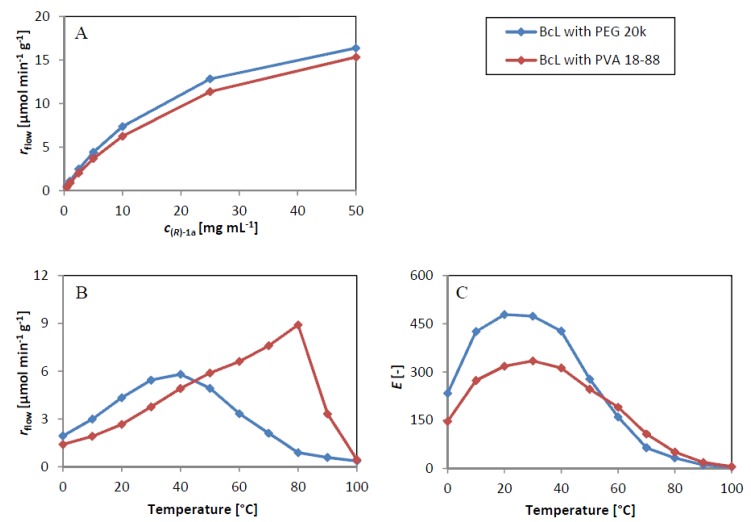
Kinetic resolutions of racemic 1-phenylethanol (*rac***-1a**) in continuous-flow packed-bed bioreactor. Effect of the substrate concentration on specific reaction rate, *r*_flow_ (**A**); temperature on specific reaction rate, *r*_flow_ (**B**) and temperature on enantiomeric ratio, *E* (**C**).

Because temperature [65] or the mode of lipase immobilization [32,66] had significant impact on lipase-catalyzed KR processes in continuous-flow reactors, two well-working adsorbed *Bc*L biocatalysts (with PVA 18–88 and PEG 20k as additives) were selected to study the effect of substrate concentration and temperature on KR of *rac***-1a** in continuous-flow packed-bed bioreactors (Figure 4).

First, productivity of the two *Bc*L biocatalysts (*r*_flow_, µmol g^−1^ min^−1^) was investigated as a function of substrate concentration (*c*_(*R*)**-1a**_, mg mL^−1^). Because the quasi-linear range of *r*_flow_ as a function of *c*_(*R*)**-1a**_ ended at 5 mg mL^−1^ (0.041 mmol mL^−1^, Figure 4A), the further temperature effect studies in the range of 0 °C–100 °C were performed at this substrate concentration (Figure 4B,C).

Next, temperature effects on productivity (*r*_flow_, Figure 4B) and selectivity (*E*, Figure 4C) of two *Bc*L biocatalysts in continuous-flow KR of *rac***-1a** were investigated between 0 °C–100 °C. In accordance with the thermal stability tests in batch mode, productivity—temperature profiles of the adsorbed *Bc*L with PEG 20k and PVA 18–88 were quite dissimilar (Figure 4B). *Bc*L with PEG 20k was thermostable only up to 30 °C and started to lose its activity over 40 °C. On the other hand, *Bc*L with PVA 18–88 was thermostable up to 80 °C and deactivated only at higher temperatures.

Investigation of the temperature-dependency of enantiomer selectivity with the two *Bc*L biocatalysts in continuous-flow KR of *rac***-1a** revealed similar trends as published previously for lipase-catalyzed KRs of secondary alcohols and amines in continuous-flow bioreactors [32,65,66]. With both forms of adsorbed *Bc*L maxima of *E* at certain temperature were found (Figure 4C). *Bc*L with PEG 20k resulted in higher enantiomer selectivity in the lower temperature range (0 °C–50 °C) with a maximum at around 20 °C but selectivity decreased drastically over the breakdown temperature of this form (~30 °C). Enantiomer selectivity of *Bc*L with PVA 18–88 had a maximum at higher temperature (~30 °C) with monotonic decrease up to 100 °C.

It was demonstrated already that operational stability of immobilized lipases in continuous-flow kinetic resolutions below the optimum temperature are quite high and stationary reaction conditions could be maintained even for one week period [51,68]. Because the main goal of this study was to demonstrate the differences between the additives and not to produce large quantities of the already known products [(*R*)-**2a** and (*S*)**-1a**], no further attempts were made for their preparative production.

*Reaction conditions*: (A) *rac***-1a** at different concentrations (0.5, 1.0, 2.5, 5.0, 10, 25, 50 mg mL^−1^) in 6/3/1 mixture of hexane/*tert*-butyl methyl ether/vinyl acetate, at 30 °C, flow rate: 0.2 mL min^−1^; (B and C) *rac***-1a** (5.0 mg mL^−1^) in 6/3/1 mixture of hexane/*tert*-butyl methyl ether/vinyl acetate, at the indicated temperature in the 0 °C–100 °C range, flow rate: 0.2 mL min^−1^. For GC analyses of samples at various states, see Experimental.

The remarkably different behavior of the two *Bc*L biocatalysts with PEG 20k and PVA 18–88 as additives in continuous-flow KR can be rationalized by assuming different solubility of the two additives. In case of PEG 20k, the additive has good solubility in the solvent (6/3/1 mixture of hexane/*tert*-butyl methyl ether/vinyl acetate) and thus dissolved out from the biocatalyst resulting in rapid loss of its positive effects. The PVA 18–88 additive in the other *Bc*L biocatalyst, however, seemed to be resistant to removal up to 80 °C. These assumptions can rationalize the enormous, about 50 °C difference in the optimum temperature of lipase activity for *Bc*L co-immobilized with PEG 20k and PVA 18–88. These results indicate also the importance of embedding the enzyme molecules in a thin and permeable matrix of suitable properties for activation and stabilization in their immobilized form.

## 3. Experimental Section

### 3.1. Analytical Methods

GC analyses were carried out on Agilent 4890 instrument equipped with FID detector using H_2_ carrier gas (injector: 250 °C, FID: 250 °C, head pressure: 12 psi, split ratio: 50:1) on a Hydrodex β-6TBDM column (25 m × 0.25 mm × 0.25 μm film with heptakis-(2,3-di-*O*-methyl-6-*O*-t-butyldimethylsilyl)-β-cyclodextrine; Macherey & Nagel, Düren, Germany). *t*_r_ (min): for **1a** and **2a** (oven: 120 °C, 8 min), 3.77 [(*S*)-**2a**], 4.08 [(*R*)-**2a**], 5.27 [(*R*)**-1a**], 5.55 [(*S*)**-1a**]; for **1b** and **2b** (oven: 100 °C–160 °C 10 °C/min), 3.87 [(*R*)**-1b**], 4.06 [(*S*)**-1b**], 4.61 [(*S*)-**2b**], 4.77 [(*R*)-**2b**]. Conversion (*c*), enantiomeric excess (*ee*) and enantiomeric ratio (*E*) were determined by GC measurements with base-line separations of the enantiomers of **1a** and **2a**, and **1b** and **2b** using precise integration methods. Enantiomeric ratio (*E*) was calculated from *c* and enantiomeric excess of the product (*ee*_P_) using the equation *E* = ln[1 − *c*(1 + *ee*_P_)]/ln[1 − *c*(1 − *ee*_P_)] [78]. Due to sensitivity to experimental errors, *E* values calculated in the 100-200 range are given as > 100, in the 200–500 range as > 200 and above 500 as » 200. To characterize the productivity of the biocatalysts, specific reaction rates (or specific biocatalyst activity) in batch reactions (*U*_b_) were calculated using the equation *U*_b_ = *n*_P_/(*t* × *m*_B_) (where *n*_P_ [μmol] is the amount of the product, *t* [h] is the reaction time and *m*_B_ [g] is the mass of the applied biocatalyst) [65]. Specific reaction rates in continuous-flow systems (*r*_flow_) were calculated using the equation *r*_flow_ = [*P*] × *v*/*m*_B_ (where [*P*] [μmol/mL] is the molar concentration of the product, *v* [mL/h] is the flow rate and *m*_B_ [g] is the mass of the applied biocatalyst) [65]. Because the rate of product formation is not a linear function of conversion (*c*), rigorous comparisons between the productivity of a continuous-flow reaction and its batch mode counterpart using their *U*_b_ and *r*_flow_ values can only be made at comparable degrees of conversions [65].

### 3.2. Materials

Lipase from *Burkholderia cepacia* (*Bc*L), phenyltrimethoxysilane (PTMOS) and (3-aminopropyl)trimethoxysilane were purchased from Aldrich (Milwaukee, WI, USA). PEG 400, PEG 1k, PEG 4k, PEG 8k, PEG 20k, gum arabic, alginic acid sodium salt from brown algae, α-cyclodextrin, β-cyclodextrin, lauric acid, decanoic acid, trilaurin, tristearin, chitosan, 1-phenylethanol were purchased from Fluka (Milwaukee, WI, USA). NaH_2_PO_4_ and Na_2_HPO_4_ were of analytical grade, purchased from Merck (Darmstadt, Germany). Hydrochloric acid (analytical grade), Brij 30, Triton X-100, PVA 4–88 (31 kDa), PVA 18–88 (130 kDa), PVA 13–23–88 (13–23 kDa), PVA 60–98 (60 kDa), PVA 72–98 (72 kDa), xylan, Tween 80, carrageenan, starch, hexanoic acid, triolein, Hymono 9004, 2-octanol, rhamnose, glucose, fructose, xylitol, xylose, sorbitol, mannitol, sucrose, maltose were purchased from Sigma-Aldrich (St. Louis, MO, USA). The mesoporous silica gel with mixed grafting [(3-aminopropyl)trimethoxysilane (ApTMOS) and phenyltrimethoxysilane (PTMOS) in mole ratio of 1:3] was the product of SynBiocat (Budapest, Hungary) [32].

### 3.3. *Bc*L Immobilization by Adsorption on Mesoporous Silica gel with Mixed Grafting

To a solution of *Bc*L (30.0 mg) in Tris buffer (14.3 mL, 100 mM, pH = 7.5, ionic strength controlled with NaCl) were added surface functionalized silica gel (300.0 mg), additive (30.0 mg) and 2-propanol (750 µL) as co-solvent. The mixture was shaken at 400 rpm at 4 °C for 24 h. The adsorbed *Bc*L biocatalyst was filtered off on a glass filter (G4), washed with distilled water (10 mL), phosphate buffer (10 mL, 20 mM, pH = 7.2), 2-propanol (2 × 10 mL), hexane (10 mL), dried at room temperature (2 h) and stored at 4 °C. All immobilization were carried out in triplicates. Standard deviations of immobilized biocatalyst masses were below 5%.

### 3.4. *Bc*L Immobilization by Adsorption Followed by Cross-Linking on Mesoporous Silica Gel with Mixed Grafting

To a solution of additive (30.0 mg) in phosphate buffer (4 mL, 20 mM, pH = 7.2) and ethanol (12 mL) was added the previously adsorbed *Bc*L preparation (200.0 mg) and glycerol diglycidyl ether (320.0 µL). The mixture was incubated at 400 rpm at 25 °C for 24 h. The adsorbed and cross-linked *Bc*L biocatalyst was filtered off on a glass filter (G4), washed with ethanol (3 × 10 mL), distilled water (10 mL), ethanol (3 × 10 mL), dried at room temperature (2 h) and stored at 4 °C. All immobilization were carried out in triplicates. Standard deviations of immobilized biocatalyst masses were below 5%.

### 3.5. Enantiomer Selective Acetylation of Racemic 1-Phenylethanol rac**-1a** and 1-(Thiophen-2-yl)ethan-1-ol rac**-1b**

Immobilized *Bc*L biocatalyst (25.0 mg; adsorbed, or adsorbed and cross-linked; with additive) was added to a solution of the racemic alcohol (*rac***-1a**: 50.0 mg; 0.409 mmol; *rac***-1b**: 50.0 mg; 0.390 mmol) in hexane/*tert*-butyl methyl ether/vinyl acetate 6/3/1 (2.0 mL) in glass vial and the resulting mixture was shaken (1,000 rpm) at 30 °C for 4 h (adsorbed *Bc*Ls) or 24 h (adsorbed and cross-linked *Bc*Ls). The reactions were analyzed by GC after 1, 2, 4 and 24 h as described in Section 3.1. All test reactions were performed in triplicates. Standard deviations of conversion were below 9%, standard deviations of enantiomeric excess were below 0.4%.

### 3.6. Thermal Stability of Immobilized *Bc*L Biocatalysts

Immobilized *Bc*L biocatalyst (25.0 mg; adsorbed, with additive) and toluene (1.0 mL) were added to 4 mL glass vial. The sample was incubated for 1 h at the given temperature (30, 50, 70, 90 or 110 °C). After cooling to room temperature, the samples were tested in KR of *rac-***1a** as described in Section 3.1.

### 3.7. Recycling the Immobilized *Bc*L Biocatalysts

Immobilized *Bc*L biocatalyst (25.0 mg; adsorbed, with additive) was added to a solution of racemic 1-phenylethanol (*rac***-1a**, 50.0 mg; 0.409 mmol) in hexane/tert-butyl methyl ether/vinyl acetate 6/3/1 (1.0 mL) in an Eppendorf tube and the resulted mixture was shaken at 1,000 rpm for 1 h at 30 °C. After 1 h, the reaction mixture was centrifuged, the immobilized *Bc*L biocatalyst was washed twice with hexane (2 × 1.0 mL), then fresh solution of rac**-1a** (50.0 mg; 0.409 mmol) in hexane/*tert*-butyl methyl ether/vinyl acetate 6/3/1 (1.0 mL) was added to it and the sample was shaken again (1,000 rpm) at 30 °C for 1 h. In this way, the immobilized *Bc*L biocatalyst were tested in 8 cycles.

### 3.8. Kinetic Resolution of 1-Phenylethanol rac**-1a** in Adsorbed *Bc*L-Filled Continuous-Flow Bioreactor

The continuous-flow KRs of *rac-***1a** were performed in a laboratory flow reactor comprising an isocratic HPLC pump (K-120, Knauer, Berlin, Germany) attached to CatCart™ columns (stainless steel, inner diameter: 4 mm; total length: 70 mm; packed length: 65 mm; inner volume: 0.816 mL) filled with the immobilized *Bc*L biocatalysts in an in–house made thermostated aluminum metal block column holder with precise temperature control. Before use, the *Bc*L-filled columns were washed with a 2:1 mixture of hexane and *tert*-butyl methyl ether (0.5 mL min^−1^, 20 min).

The adsorbed *Bc*L biocatalysts (on mesoporous silica gel with mixed grafting in the presence of PVA 18–88 or PEG 20k) were packed into stainless steel CatCart™ columns according to the filling process of ThalesNano (Budapest, Hungary). Before packing, the *Bc*L biocatalyst-filled columns were washed with distilled water, ethanol, *n*-hexane and acetone in an ultrasonic cleaner. For the continuous-flow enzymatic applications, the columns were sealed by silver metal filter membranes [Sterlitech Silver Membrane Filter from Sigma–Aldrich, Z623237, pore size 0.45 μm; pure metallic silver, 99.97% with no extractable or detectable contaminants] due to the known benefits of Ag (bacteriostatic). The sealings were made of PTFE. Two CatCart™ columns per enzyme were packed for this study (filling weights: *Bc*L with PVA 18–88, 237.1 mg and 237.7 mg; *Bc*L with PEG 20k, 233.6 mg and 245.9 mg).

To study the effect of the substrate concentration, solutions with racemic 1-phenylethanol (*rac***-1a**) at different concentrations (0.5, 1.0, 2.5, 5.0, 10, 25, 50 mg mL^−1^) in 6/3/1 mixture of hexane/*tert*-butyl methyl ether/vinyl acetate were pumped through the adsorbed *Bc*L biocatalyst-filled columns (adsorbed *Bc*L with PVA 18–88 or PEG 20k) thermostated to 30 °C at a flow rate of 0.2 mL min^−1^. At each concentration, samples were analyzed by GC every 10 min up to 40 min after the beginning of the experiment. Samples were collected during stationary operation (30 min after changing the parameters), diluted with EtOH to 2 mg mL^−1^ and analyzed as described in Section 3.1.

To study the effect of the temperature, a solution of racemic 1-phenylethanol (*rac***-1a**, 5.0 mg mL^−1^) in 6/3/1 mixture of hexane/*tert*-butyl methyl ether/vinyl acetate was pumped through the adsorbed *Bc*L biocatalyst-filled columns (adsorbed *Bc*L with PVA 18–88 or PEG 20k) thermostated to various temperatures (0 °C–100 °C) at a flow rate of 0.2 mL min^−1^. Samples were collected during stationary operation (30 min after changing the parameters) and analyzed as described above. The experiments were performed at 10 °C steps in the temperature range between of 0 °C–100 °C.

After performing the various tests, the adsorbed *Bc*L biocatalyst-filled columns were washed with a 2:1 mixture of hexane and *tert*-butyl methyl ether (0.5 mL min^−1^, 20 min) and stored at 4 °C.

## 4. Conclusions

Catalytic properties and stability of lipase from *Burkholderia cepacia* could be tuned effectively with additives in immobilization on mixed-function-grafted silica gel support by hydrophobic adsorption and covalent attachment.

Tests with the immobilized *Bc*Ls using kinetic resolution of *rac***-1a** and *rac***-1b** revealed *Bc*L adsorbed in the presence of high molecular weight polymeric additives was significantly more active than adsorbed *Bc*L without additive. The highest specific enzyme activity with PEG 20k as additive and with PVAs can be rationalized by assuming a thin film of the polymeric additive embedding the *Bc*L molecules. Most of the investigated additives had a positive impact on covalent immobilization of *Bc*L with cross-linking by glycerol diglycidyl ether after adsorption and resulted immobilized *Bc*Ls with moderate activity. Cross-linking without additives was fatal to *Bc*L activity. Even the additive-protected cross-linked *Bc*Ls exhibited only about tenfold lower activity than their non-cross-linked counterparts.

Due to the good mechanical properties of the carrier, most of the adsorbed *Bc*Ls were well recyclable from their reactions in organic media and maintained their catalytic activity up to 8 runs. Thermal stability of *Bc*Ls adsorbed with PEG 20k and PVA 18–88 in batch and in continuous-flow systems were remarkably different. Adsorbed *Bc*L with PEG 20k lost its activity above 40 °C, however *Bc*L with PVA 18–88 remained stable up to 80 °C with a selectivity maximum at approximately 30 °C. The remarkable 50 °C shift of optimum temperature of lipase activity for *Bc*L co-immobilized with PEG 20k (at around 30 °C) and *Bc*L co-immobilized with PVA 18–88 (at around 80 °C) clearly indicate the potential of “immobilization engineering” for tuning the properties of immobilized enzymes.
